# Movement kinematics and proprioception in post-stroke spasticity: assessment using the Kinarm robotic exoskeleton

**DOI:** 10.1186/s12984-019-0618-5

**Published:** 2019-11-21

**Authors:** George Mochizuki, Andrew Centen, Myles Resnick, Catherine Lowrey, Sean P. Dukelow, Stephen H. Scott

**Affiliations:** 1 Heart and Stroke Foundation Canadian Partnership for Stroke Recovery, Sunnybrook Research Institute, Toronto, Ontario Canada; 2 Hurvitz Brain Sciences Research Program, Sunnybrook Research Institute, Toronto, Ontario Canada; 3 Department of Physical Therapy, Faculty of Medicine, University of Toronto, Toronto, Ontario Canada; 4 Toronto Rehabilitation Institute, University Health Network, Toronto, Ontario Canada; 5 Rehabilitation Sciences Institute, University of Toronto, Toronto, Ontario Canada; 6 School of Kinesiology and Health Science, Faculty of Health, York University, 4700 Keele St, Bethune College Rm 363, Toronto, Ontario M3J1P3 Canada; 7 Centre for Neuroscience Studies, Queen’s University, Kingston, Ontario Canada; 8 Department of Clinical Neurosciences, Hotchkiss Brain Institute, University of Calgary, Calgary, Alberta Canada; 9 Department of Biomedical and Molecular Science, Queen’s University, Kingston, Ontario Canada

**Keywords:** Stroke, Spasticity, Upper extremity, Kinematics, Robotics

## Abstract

**Background:**

Motor impairment after stroke interferes with performance of everyday activities. Upper limb spasticity may further disrupt the movement patterns that enable optimal function; however, the specific features of these altered movement patterns, which differentiate individuals with and without spasticity, have not been fully identified. This study aimed to characterize the kinematic and proprioceptive deficits of individuals with upper limb spasticity after stroke using the Kinarm robotic exoskeleton.

**Methods:**

Upper limb function was characterized using two tasks: Visually Guided Reaching, in which participants moved the limb from a central target to 1 of 4 or 1 of 8 outer targets when cued (measuring reaching function) and Arm Position Matching, in which participants moved the less-affected arm to mirror match the position of the affected arm (measuring proprioception), which was passively moved to 1 of 4 or 1 of 9 different positions. Comparisons were made between individuals with (*n* = 35) and without (n = 35) upper limb post-stroke spasticity.

**Results:**

Statistically significant differences in affected limb performance between groups were observed in reaching-specific measures characterizing movement time and movement speed, as well as an overall metric for the Visually Guided Reaching task. While both groups demonstrated deficits in proprioception compared to normative values, no differences were observed between groups. Modified Ashworth Scale score was significantly correlated with these same measures.

**Conclusions:**

The findings indicate that individuals with spasticity experience greater deficits in temporal features of movement while reaching, but not in proprioception in comparison to individuals with post-stroke motor impairment without spasticity. Temporal features of movement can be potential targets for rehabilitation in individuals with upper limb spasticity after stroke.

## Background

Sensorimotor impairments after stroke result in functional deficits that are targets for neurorehabilitation interventions. Important to effective implementation of these interventions is an understanding of the characteristics of the specific deficits that persist after stroke. Better alignment between these specific deficits and the rehabilitation approach may enhance opportunities for recovery after stroke.

The impairments that manifest after stroke generally reflect abnormal synergy patterns or reduced (i.e. weakness/paresis) or exaggerated (i.e. spasticity) motor activity. Indeed, individuals with spasticity, defined as a motor disorder characterized by a velocity-dependent increase in stretch reflexes resulting from hyperexcitability of the stretch reflex [1], can demonstrate involuntary activation of muscles [2], soft-tissue contracture, and muscle overactivity [3]. Reductions in spasticity can increase use of the affected limb [4] and improve functional outcomes [5–8], though the mechanism of improvement (i.e. enhanced proprioception, normalized kinematic patterns) is not well established. Determining the features (i.e. components) of movement that are impaired in individuals with spasticity may subsequently identify potential targets for therapeutic interventions, which may facilitate recovery. As a first step, it is necessary to characterize sensorimotor impairment in individuals with post-stroke spasticity during active functional tasks.

A recent systematic review reported that a moderate improvement in activity performance or capacity (within the context of the International Classification of Functioning, Disability and Health (ICF) framework) occurs with reductions in spasticity [6]. Reductions in spasticity are associated with improvements on the Lindmark Motor Assessment Scale [9], amount-of-use and quality-of-movement scores of the Motor Activity Log [4], Goal Attainment Scaling [10], and tasks such as hand hygiene and dressing [11, 12]. In contrast, reductions in spasticity have no effect on the Action Research Arm Test [4, 11] or the Box and Block Test [4]. One possible factor contributing to the variability in these findings is that these outcome measures are not constructed to characterize the features of movement that contribute to the specific deficit. In contrast, robotic technologies may provide information on the specific features of functional movement that are impaired after stroke [13–17]. For example, Bosecker, Dipietro, Volpe, and Krebs (2010), demonstrated that performance on kinematic measures were predictors of clinical outcomes [18]. In addition, the Kinarm robotic exoskeleton has been used as a probe of upper limb function using a Visually Guided Reaching (VGR) task to probe postural and motor control [16], an object hit task to probe bimanual sensorimotor performance [15], and a limb-position matching task to probe multi-joint limb position sense [17]. Given the apparent sensitivity of these tasks to quantitatively measure impairment in upper limb function and proproprioception after stroke, they may also be useful in characterizing the features of motor and proprioceptive impairment that are unique to individuals with spasticity.

The objective of this study was to characterize the features of kinematics and proprioception that are impaired in individuals with upper limb spasticity after stroke using the Kinarm robotic exoskeleton. The two tasks performed in the study were the VGR task and the Arm Position Matching (APM) task. VGR was included because it requires fast, co-ordinated reaching movements to stationary targets, and thus is relevant to performance of some everyday tasks. The APM task was used to assess proprioception, which is integral for body image and planning motor actions. It was hypothesized that more severe deficits in measures of movement kinematics and limb proprioception would both be observed in post-stroke individuals with clinically-identified spasticity compared to post-stroke individuals without spasticity.

## Methods

### Participants

Individuals with stroke were recruited from the Toronto Rehabilitation Institute and Sunnybrook Health Sciences Centre in Toronto, Canada, the inpatient acute stroke unit and stroke rehabilitation units at Foothills Medical Centre and the inpatient stroke rehabilitation units at Dr. Vernon Fanning Care Centre in Calgary, Canada and St. Mary’s on the Lake or Providence Care Hospital in Kingston, Canada. Participants were included in the study if they were over 18 years of age, had a confirmed diagnosis of stroke, could understand the task instructions, were able to maintain a position of 90° shoulder abduction with support, had normal or corrected vision, and were able to participate in the informed consent process. Individuals were excluded if the assessments could be influenced by a pre-existing neurological condition, cognitive/behavioural issue, or a communication limitation. All participants provided informed consent prior to participation in the study. All procedures and methods were approved by the ethics boards of the Toronto Rehabilitation Institute, Sunnybrook Health Sciences Centre, and the University of Toronto, the Queen’s University Health Sciences and Affiliated Teaching Hospitals Research Ethics Board (#ANAT042–05), and the University of Calgary’s Conjoint Health Research Ethics Board (#22123).

Presence of elbow spasticity was assessed by a physiotherapist or a trained study investigator using the Modified Ashworth Scale [19] (MAS ≥ 1 indicating the presence of spasticity). The Chedoke McMaster Stroke Assessment (CMSA, [20]) arm subscale was implemented by a physical or occupational therapist at the time of enrollment into the study. In some instances, CMSA was retrospectively collected from the participants’ admission to inpatient services and used as an indicator of impairment. The CMSA uses a 7-point scale reflecting stages of motor recovery following stroke (7–highest recovery stage, 1–lowest recovery). Affected side of stroke participants was determined clinically as the most affected side of their body. We refer to the other side of the body as the “less-affected” side, as ~ 30% of individuals with stroke experience impairment in the arm ipsilateral to the lesioned hemisphere [16, 21].

### Experimental setup

**Fig. 1:**
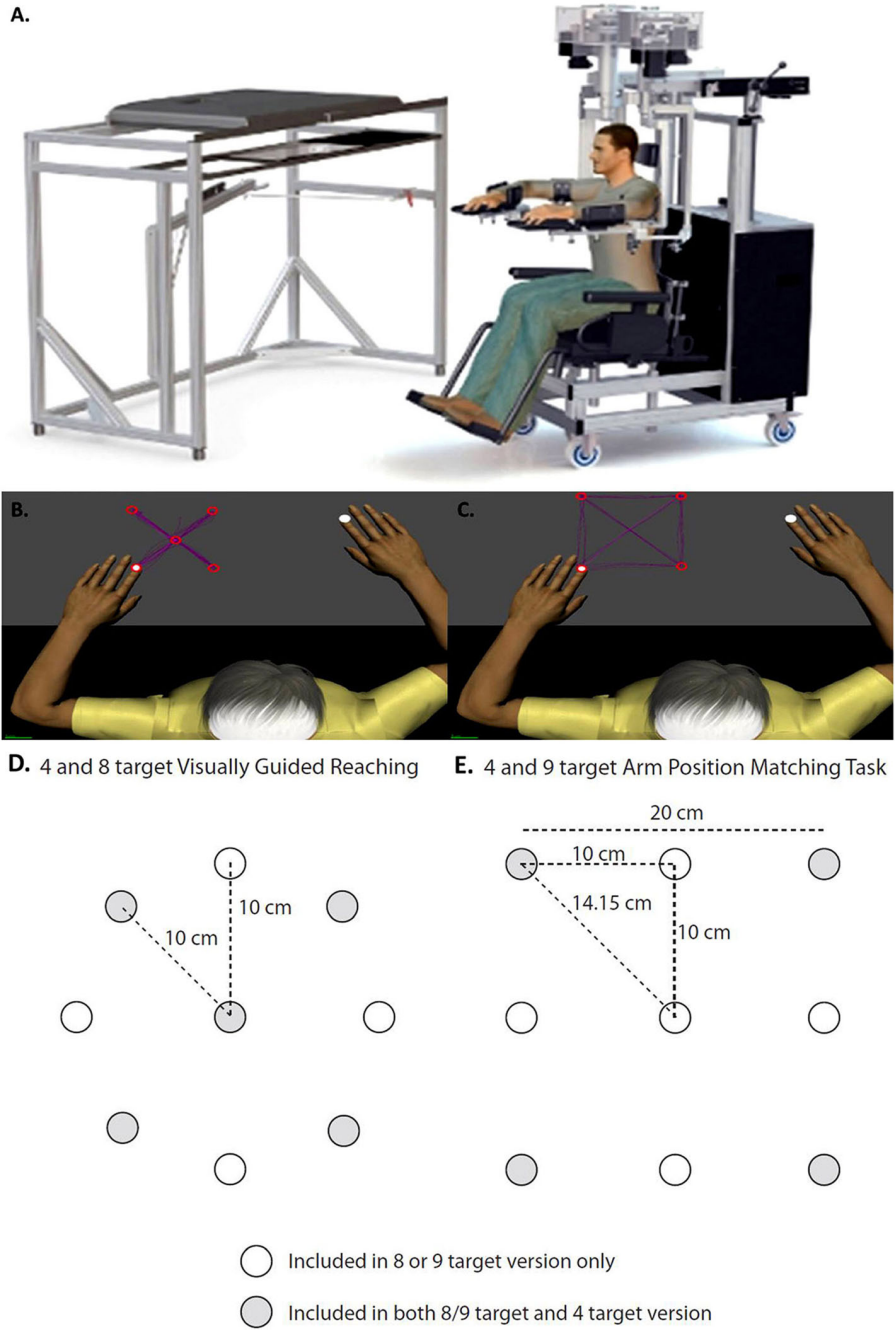
**a** Diagram of the Kinarm robotic exoskeleton. Schematic representations of the tasks included in the present study, including: **b** Visually Guided Reaching from a central fixation point to 4 randomly presented targets; **c** Arm Position Matching of one limb to one of 4 targets to which the opposite limb is moved; **d** Schematic representation of the target locations for the 4 and 8 target Visually Guided Reaching task; **e** Schematic representation of the target locations for the 4 and 9 target Arm Position Matching task. In **d** and **e**, the white circles depict the targets included in the 8 or 9 versions only and the grey circles depict the targets included in both the 8/9 target and 4 target versions

A detailed description of the Kinarm robotic exoskeleton for the upper limb (Kinarm, Kingston, Canada) has been presented previously [15–17]. The Kinarm robot collects shoulder and elbow kinematic information during tasks performed in the horizontal plane and can apply loads to move the arm in the workspace. Participants are seated with shoulders abducted ~ 85° and arms resting in troughs with full weight support of the limbs (Fig. 1a). Linkages of the robot are aligned with the actual joints of the participant. Calibration procedures were carried out for each participant and included locating fingertip position, defining a known elbow angle, and measuring segment lengths for both arms. All tasks were controlled and relayed using a real-time computer and Dexterit-E™ (versions 2.3.0–3.6.4) data acquisition software. During each task, participants interact with a 2-D virtual reality display unit where task objects appear on the same horizontal plane as the participant’s arms.

Detailed descriptions of the tasks used in this study have been reported previously. These include: Visually Guided Reaching (VGR – 4 or 8 target version )[16] and Arm Position Matching (APM – 4 or 9 target versions )[17]. The 4 target versions of the task were developed from the original 8 and 9 target versions to shorten the duration of the task and use a subset of the original targets. During the VGR task, the participant reached from a central target to one of four or eight randomized peripheral targets as quickly and accurately as possible (Fig. 1b). Each target was presented five times for the four target version and eight times for the eight target version of the reaching task. VGR was assessed on both the affected and less-affected limbs. During the APM task, vision of the limbs was blocked and the robot moved the affected limb to one of four or nine randomized positions in the workspace. The participant was asked to mirror-match the position of the limb with the opposite arm. Once the participant informed the operator that the movement was completed (i.e. they had perceived that they had matched the position), the robot was prompted to move the limb to another position in the workspace (Fig. 1c). This was repeated until all four positions were attempted five times for the four target version and six times for the nine target version of the task. APM was assessed for the less-affected limb only (i.e. robot moved the affected limb) to avoid the issue of separating sensory and motor impairment if the affected limb was required to position match (i.e. if the robot moved the less-affected limb). The differences in target location for the 4, 8, and 9 target versions are presented in Fig. 1d and e.

### Outcome measures

The outcome measures from each task were selected because they represented different components of sensorimotor control including speed, stability, smoothness, error correction, and proprioception [16, 17]. In total, nine outcome measures were used for the less-affected limb and six measures were used for the affected limb. These included:

1. Visually Guided Reaching task (VGR)
  - Posture Speed (PS) – A descriptor of the individual’s ability to keep the hand steady at the central target. This was calculated as the median hand speed for 500 ms prior to presentation of the peripheral target. The median of all trials is calculated as the overall posture speed.
  - Initial Direction Angle (IDA) – Angular deviation between a straight line from the initial hand position and the hand position after the initial phase of movement compared to a straight line from the initial hand position to the destination target. The initial phase of movement is defined as the time from movement onset to the first speed minimum after movement onset. Movement onset is identified by determining when the hand first exits the start target after the end target is illuminated and then searching back in time to determine a point where the hand speed dips below the maximum calculated posture speed. If this point cannot be determined using this algorithm, then movement onset is set as the first time the subject left the start target after illumination of the end target.
  - Speed Maxima Count (SMC) – A measure of smoothness determined by counting the number of speed peaks from movement onset to movement termination.
  - Movement Time (MT) – Time between movement onset and movement termination. This was included as a general descriptor of movement.
  - Path Length Ratio (PLR) – A ratio of the length of the total movement relative to the length of a straight line between initial position and target.
  - Maximum speed (MS) – Peak speed of the movement.

2. Arm Position Matching task (APM)

- Variability (Var) – an indicator of the trial-to-trial consistency of the active hand. Variability was calculated for each target location as the standard deviations of the subject’s hand position in both the X and Y directions (Var_x_ and Var_y_). Variability XY was calculated as follows:
  $$ Variability\  XY=\sqrt{{{\mathit{\operatorname{var}}}_x}^2+{{\mathit{\operatorname{var}}}_y}^2\ } $$

b. Spatial Shift (Shift) – indicator of systematic errors between the active and passive hands. This was calculated as the mean error between the active and passive hands for each target location, and then the mean of means for all target locations. Systematic shifts were calculated in the x (shiftx) and y (shifty) directions. Combined shift in both x and y was calculated as follows:
  $$ Shift\  XY=\sqrt{{shift_x}^2+{shift_y}^2\ } $$

c. Contraction/Expansion ratio (Con/Exp XY) – indicator of the area of the workspace comprising the outer 4 or 8 targets ‘matched’ by the active hand in comparison to that of the passive hand. This was determined by calculating the area of movement of the active hand and normalizing it by the area covered by the passive hand.
  $$ Con/ Exp\  XY=\frac{area_{xy\_ active}}{area_{xy\_ passive}} $$

To compare parameters between groups, standardized Z-scores were calculated for each parameter using Dexterit-E software (Analysis Version 3.7). Parameter scores were compared to a large cohort of healthy control data (VGR: *N* = 288 participants, 18–84 years old, 127 males; APM: 799 participants, 18–93 years old, 363 males) available through the Dexterit-E Analysis software. Details of this process have been outlined previously [14, 22] and online (https://kinarm.com/kinarm-products/kinarm-standard-tests). Briefly, control data were normalized using Box-Cox transformations. The data were fit using multiple linear regression (MLR) to account for age, sex and handedness. Box-Cox equations were adjusted if necessary to attain a normal distribution and Z-scores were calculated for normal or transformed to normal parameters. Z-scores were calculated for participants with stroke using the same parameter models developed from the healthy control participant data. Standard cut-off scores were used to determine whether performance of individual participants with stroke fell outside of the normative bounds. For a one-sided comparison where a larger parameter value reflected poor performance (i.e. posture speed) the cut-off of Z = 1.65 was used (95th percentile). For a one-sided comparison where a smaller parameter value reflected poor performance (i.e. maximum speed) the cut-off of Z = − 1.65 was used. For two-sided comparisons where either extreme reflects poor performance (i.e. contraction/expansion ratio) Z = 1.96 or − 1.96 cutoffs were used (2.5th, 97.5th percentiles).

**Fig. 2:**
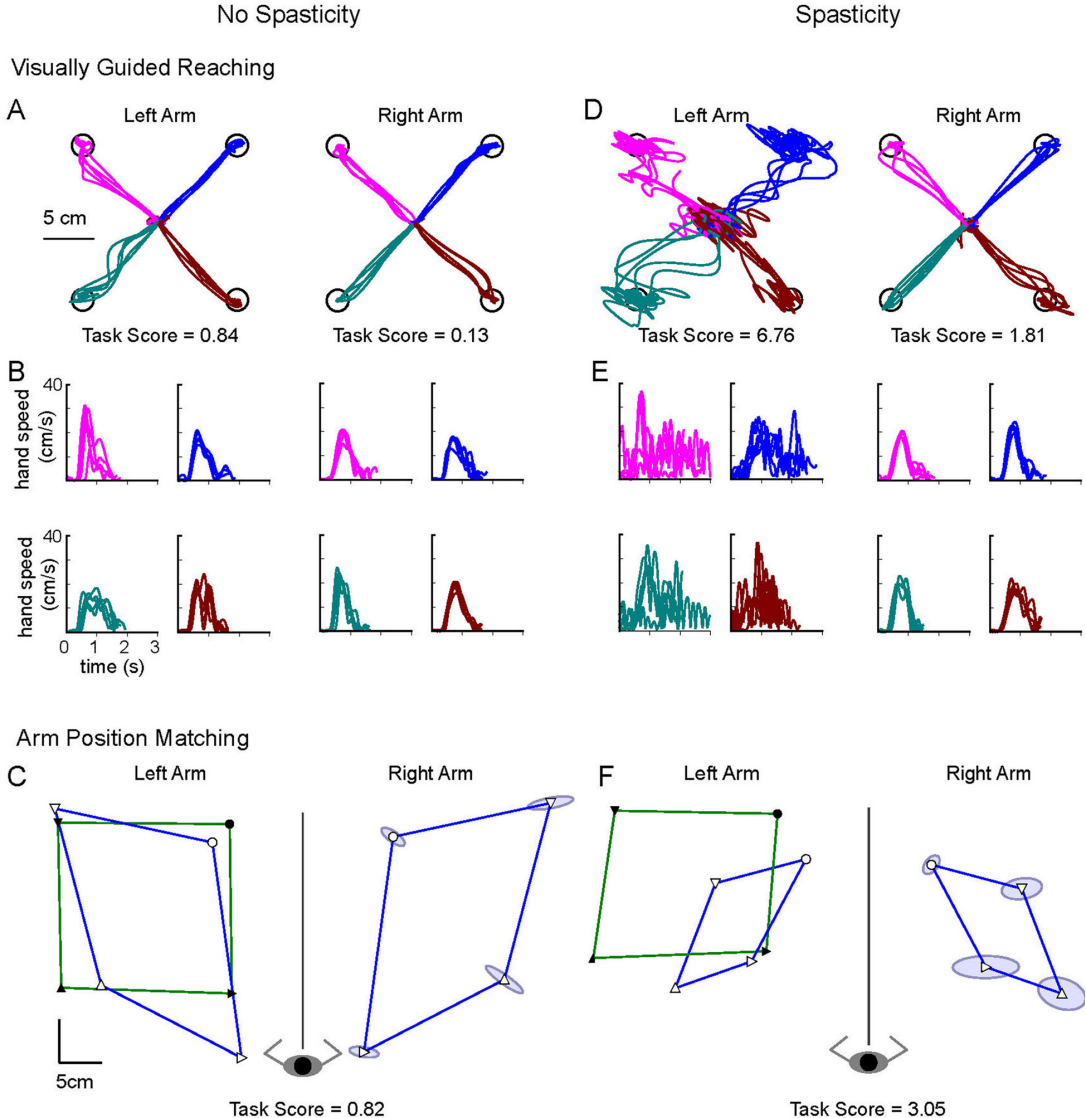
Task Performance of two exemplar participants. **a**-**c** Participant from the No Spasticity group: Female, Right-handed, 70 years old, 7 months post-stroke, Left-Affected, MAS of 0, CMSA arm (at intake) of 3. **d**-**f** Participant from the Spasticity group: Female, Right handed, 35 years old, 6 months post-stroke, Left-Affected, MAS of 1+, CMSA arm (at intake) of 3. **a** and **d** show the hand traces for the Visually Guided Reaching task. Only the reaches out to the target are shown. B and E show the hand speeds for the reaches out to each target. Colour scheme matches the traces in **a** and **d**. **c** and **f** reflect the performance on the Arm Position Matching task where the robot moved the affected left arm to four locations (solid symbols – green line represents the perimeter of the targets) and the participant matched the position with the less-affected right arm (open symbols - blue line represents the perimeter of the targets). Matching performance is mirrored and displayed over the left side for comparison purposes. Ellipses around the icons reflect the spatial variability (1 standard deviation) of all matching trials at that target position. Task Scores are shown below each (Task Score > 1.96 indicates that performance fell outside 95% range of healthy control behaviour)

To further characterize performance on each task in the context of healthy behaviour, ‘failure’ on each task was determined by deriving the Task Score [22]. Briefly, the Task Score is derived from a root sum of squares (RSS) of all the healthy participant Z-score values for all parameters from a given task. The RSS values are then transformed to normal using Box-Cox equations [23] and further transformed to a Task Score such that 0 equals best performance and poor performance is reflected by higher values. Task Scores were calculated for participants with stroke using the same parameter models developed from the control participant data. Because the Task Scores are based on Z-scores calculated relative to a healthy control dataset, a Task Score > 1.96 on for the VGR or APM reflects performance outside of the 95% confidence limit for healthy age-matched individuals on that task. Therefore, this cutoff was used to quantify the proportion of individuals failing each task. Figure 2 depicts reaching trajectories and matching ability for 2 representative participants (with and without spasticity).

### Statistical analyses

Descriptive statistics were used to characterize the study groups: individuals with spasticity (Spasticity) and individuals without spasticity (No Spasticity). Wilcoxon rank sum tests were used to determine whether individuals with spasticity who were or were not taking anti-spastic medication differed on any of the measures. Selected parameters from the robotic tasks were extracted from standardized reports generated by the Dexterit-E software. To test the hypothesis that individuals with spasticity would demonstrate greater deficits than individuals without spasticity, Kolmogorov-Smirnov tests were used to compare parameter Z-scores. Pearson’s Chi-Square was used to determine whether the proportion of participants in a group that failed a task (Task Score > 1.96) differed from the proportion of participants who were within normative bounds. Spearman’s correlations were conducted to determine the level of association between the MAS scores and parameter or task scores for the affected limb (VGR task only) and less-affected limb (VGR and APM tasks). Analyses were conducted using SPSS v23 (IBM, Armonk, USA) and Matlab (Mathworks, Natick, USA). The alpha level for statistical significance was set at *p* ≤ 0.05 and all tests were corrected for multiple comparisons using Bonferroni corrections. Adjusted *p*-values are reported.

## Results

A total of 70 individuals with stroke were included in the study. Thirty five participants were included in each of the Spasticity and No Spasticity groups. Critically, we matched participants in terms of CMSA scores at the time of admission in an attempt to match the initial level of impairment between the two groups (Table 1). All participants with spasticity scored MAS ≥1 on the elbow flexors. Four individuals with spasticity were being treated with antispastic medication (baclofen, benzodiazapines). Seven others were assessed at a time point > 90 days after focal injection with onabotulinum toxin. A comparison of all measures between all individuals with spasticity who were (*n* = 11) or were not (*n* = 24) receiving anti-spastic medications revealed statistically significant differences in CMSA (median CMSA = 4 and CMSA = 3, medication vs non-medication, respectively; z = 2.54, *p* = 0.02) and Time post stroke (21 months vs 6 months, medication vs non-medication, repsecitvely, z = 2.30, *p* = 0.01). No differences were found between the medication vs non-medication groups for any parameter Z-score or Task Score so data were grouped. Demographics and clinical information for all enrolled participants are presented in Table 1. Time post-stroke denotes the time when the Kinarm assessment was performed.

**Table 1:** Participant Information

	No Spasticity (*N* = 35)	Spasticity (*N* = 35)
Age (years)^a^	62.8 (27–87)	56.5 (18–78)
Sex (M/F)	25/10	24/11
Handedness pre-stroke (L/R/A)	3/31/1	3/32/0
Affected side of body (L/R)	16/19	20/15
Time post stroke (months)^a^	6.28 (1–14.5)	14.73 (2–154)
Time to intake (days)^a^	13.7 (4–34)	19.7 (2–39)
CMSA affected arm (at intake)^b^	3 (2–5)	3 (2–5)
Scores:	[2 3 4 5]	[2 3 4 5]
# of participants:	[4 11 15 5]	[4 11 15 5]
MAS (flexors, at time of robotic assessment)^b^	0	1.5 (1–3)
Scores:		[1 1+ 2 3]
# of participants:		[13 10 9 3]

Abbreviations: *MAS* Modified Ashworth Scale, *CMSA* Chedoke-McMaster Stroke Assessment (arm), ^a^data presented as mean (range); ^b^data presented as median (range). The median MAS score of 1.5 represents an actual score of 1+. Time to intake refers to the amount of time between the stroke and CMSA testing, which was completed at their intake into clinic

By observation, many participants in both groups demonstrated deficits in both the VGR and APM tasks. For the VGR tasks, these deficits were manifested as trajectory errors, limitations in range of motion, movement during intended periods of fixation on a target, and limitations in target accuracy involving the affected arm. For the APM tasks, the deficits were observed in the extent of trial-to-trial variability, spatial shift, and area of the workspace covered by the less-affected arm. Figure 2 presents exemplar performance data for both tasks for individuals in both groups.

**Fig. 3:**
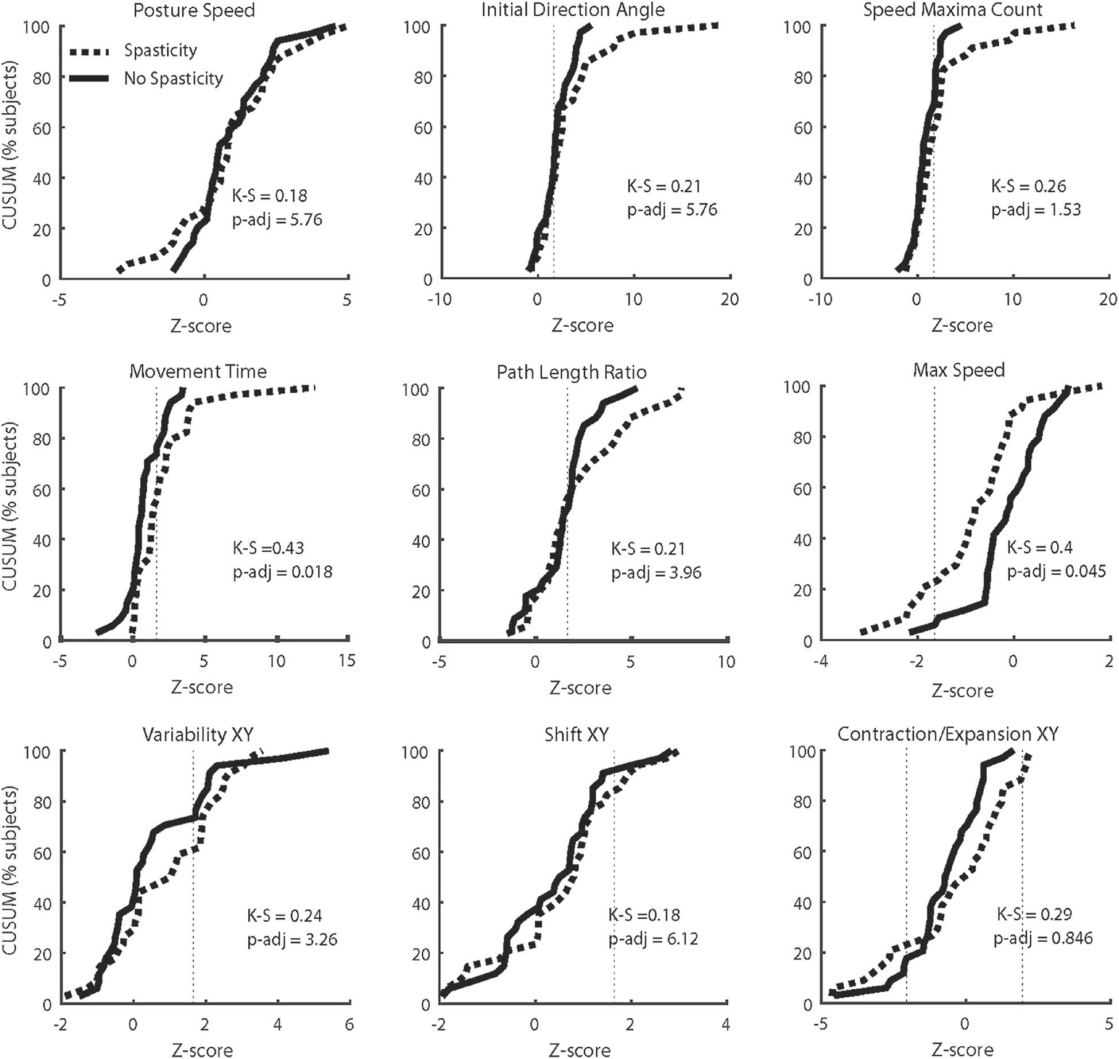
CUSUM (Cumulative Sum) plots for each outcome measure demonstrating the proportion of individuals from the Spasticity group (dashed lines) and the No Spasticity group (solid lines) who fail each task. A ‘fail’ is counted as a score exceeding the upper bound of the 95% limit of the range of normal healthy controls (dashed vertical line). A ‘fail’ on Contraction/Expansion XY was a score above or below the 95% limit of the range of normal healthy controls. The output from the Kolmogorov-Smirnov tests and adjusted *p* values are presented on each panel

In general, a proportion of participants in each group had deficits on each parameter (Fig. 3; Table 2). A higher percentage of participants in the Spasticity group were identified as impaired on almost every parameter tested (except Path Length Ratio for VGR) compared to the No Spasticity group. Direct comparisons of parameter distributions identified statistically significant differences in Movement Time (KS = 0.43, p-adj = 0.018) and Maximum Speed (KS = 0.40, p-adj = 0.045) (Fig. 3). There were no differences between groups for APM task parameters (Fig. 3).

**Table 2:** Parameter scores, Z scores, Task scores, and the proportion of participants from each group failing each parameter. A ‘fail’ is identified as a score falling outside of the 95% Confidence Interval of healthy controls

	Parameter data (median, range)		Z-score (median) & Task score (mean)		Proportion failed (%)	
Parameter	No Spasticity	Spasticity	No Spasticity	Spasticity	No Spasticity	Spasticity
VGR						
Posture Speed (m/s)	0.005 (0.003–0.018)	0.005 (0.001–0.026)	0.53	0.82	29	32
Initial Direction Angle (rad)	0.069 (0.037–0.211)	0.07 (0.033–1.20)	1.77	1.93	62	65
Speed Maxima Count	2.56 (1.65–4.46)	2.83 (1.97–11)	0.58	1.16	32	41
Movement time (s)	1.11 (0.70–1.72)	1.27 (0.96–4.20)	0.59	1.32	24	44
Path Length Ratio	1.22 (1.06–1.74)	1.22 (1.06–2.92)	1.70	1.5	53	47
Max Speed (m/s)	0.24 (0.14–0.32)	0.21 (0.13–0.33)	−0.09	− 0.61	3	21
VGR-affected Task Score	–	–	2.09	3.07	50	76
VGR-less affected Task Score	–	–	0.91	1.30	18	24
APM						
Variability XY (m)	0.036 (0.023–0.13)	0.041 (0.021–0.11)	0.09	0.93	29	38
Shift XY (m)	0.053 (0.007–0.172)	0.058 (0.007–0.15)	0.50	0.71	9	15
Con/Exp XY	0.67 (0.047–1.25)	0.841 (0.036–1.40)	−0.72	−0.37	18	29
APM Task Score	–	–	1.49	1.78	24	41

Based on the 95% confidence limits (Task Scores), a proportion of participants failed each task. For the VGR task with the affected limb, 76 and 50% of individuals in the Spasicity and No Spasticity groups, respectively, failed the task. These proportions were 24 and 18% for the same groups with the less-affected limb. The Chi-square analysis revealed statistically significant differences in the proportion of individuals failing the VGR-affected limb between groups (χ^2^(1) = 5.044, *p* = 0.025). No statistically significant difference in proportion was observed for the VGR-less affected (χ^2^(1) = 2.365, *p* = 0.124). For APM, the proportion of individuals in the Spasicity and No Spasticity groups failing the task with the affected limb was 41 and 24%, respectively. The Chi-square analysis revealed no statistically significant differences in the proportion of individuals failing the APM task (χ^2^(1) = 0.0899, *p* = 0.7642).

**Fig. 4:**
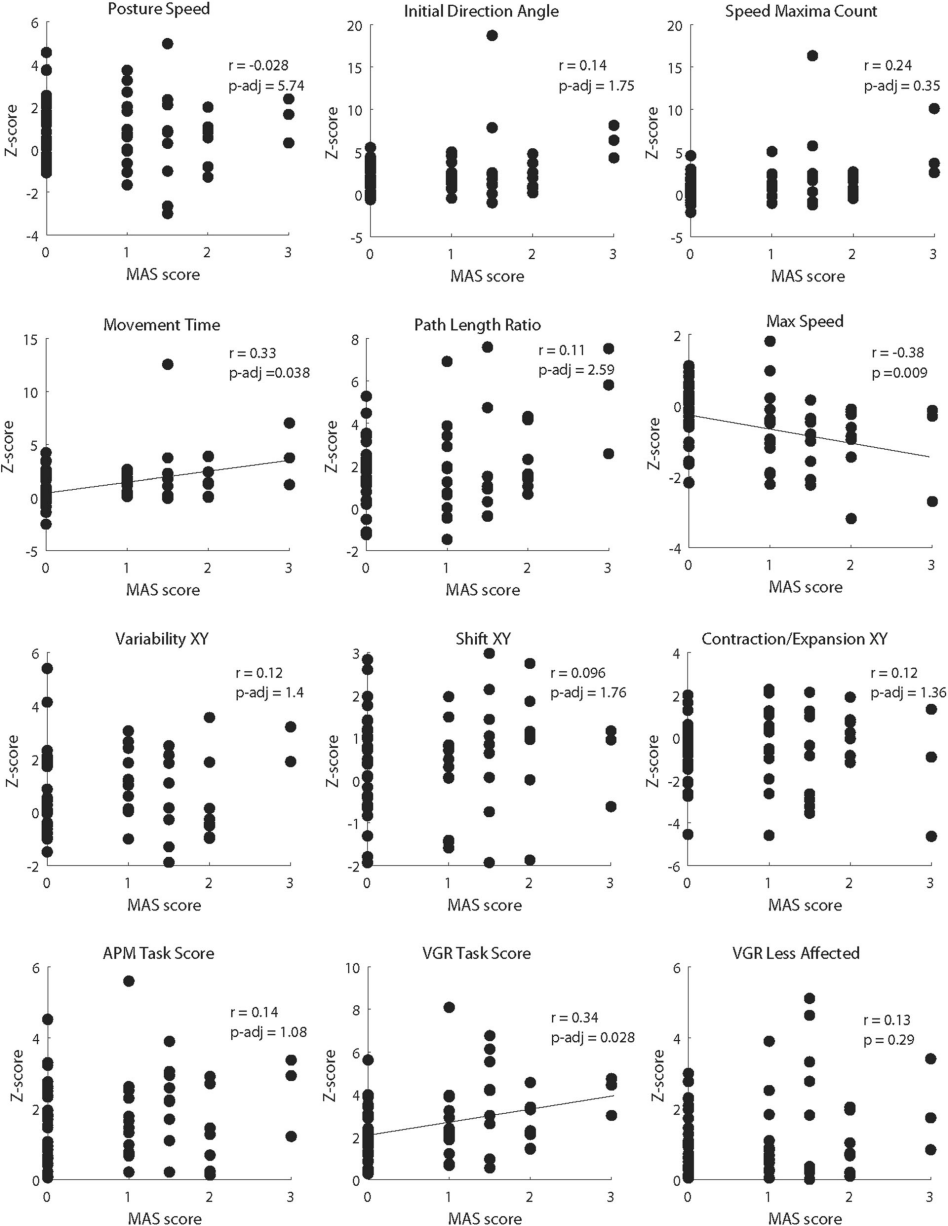
Scatterplots showing the relationship between MAS score and each outcome measure (including Task Scores) for the Visually Guided Reaching and Arm Position Matching tasks. Spearman’s r and the adjusted *p* value for each correlation are presented on each graph. Lines of best fit are included on those graphs in which a statistically significant correlation between outcome measure and MAS was observed

Spearman’s correlation coefficients were generated to quantify the strength of association between each of the outcome measures and MAS assessed for the flexors (Fig. 4). This analysis identified modest but statistically significant correlations between MAS and Movement Time (r = 0.33, p-adj = 0.038), Maximum Speed (r = − 0.38, p-adj = 0.009) and VGR Task Score (r = 0.34, p-adj = 0.028).

## Discussion

The objective of this study was to characterize the features of movement kinematics and proproprioception that are impaired in individuals with upper limb spasticity after stroke, when controlling for the initial level of impairment. The analyses identified that individuals with spasticity demonstrate greater deficits in features of motor function related to movement time and movement speed, as well as an overall metric of motor function. These measures were also associated with spasticity. In contrast, although a higher proportion of people with stroke (with or without spasticity) demonstrated deficits in proprioception compared to established normative values, none of the measures of proprioception differed between groups. The findings provide evidence indicating that specific features of motor control, especially those associated with temporal features of movement tend to be more impaired in individuals with upper limb spasticity after stroke.

### Visually guided reaching – errors in temporal features of motor function

Individuals with spasticity demonstrated greater deficits in outcome measures for the VGR task measuring the temporal features of movement. In addition, MAS was low-to-moderately correlated with the same two outcome measures. These findings point to the presence of spasticity as being associated with deficits in features of upper limb motor control related to movement timing. The important clinical consideration here is that, in the context of these motor assessments, spasticity is linked to the time required to perform a task and the speed at which a task can be performed. The present findings align with prior work demonstrating that movement time [24] and speed are associated with the presence and/or severity of spasticity and that peak movement speed is lower in individuals with spasticity prior to the onset of spasticity management with botulinum toxin in comparison to healthy controls [4]. Individuals with spasticity demonstrate an ability to increase reaching speed [25]; however, to be able to do this, compensatory strategies are used (i.e. increased trunk motion if the trunk is unconstrained). In the current experiment, the exoskeleton would have limited the occurrences of compensatory movements. As a result, individuals with spasticity would have relied on their existing capacity for movement at the shoulder and elbow in the absence of assistance from compensatory strategies. Consequently, the challenge of overcoming higher flexor tone may have induced impediments in both the time required to perform the task and the speed at which the task could be performed.

Slowing of movement may also reflect a learned strategy to maximize task performance as motor learning capacity persists in individuals with stroke [26]. However, Subramanian, Feldman, and Levin [27] reported that spasticity may hinder motor learning capacity after stroke, especially if the angular position of the elbow while learning the task is within a spatial ‘spasticity zone’ – the angular range within which spasticity is observed. The larger deficits in temporal metrics observed in our spastic cohort may have occurred at elbow positions that were within the range of the spastic zone. Deficits in inter-joint coordination [24] (i.e. between shoulder and elbow) in the spastic cohort may also contribute to greater detriments in movement time and movement speed. The VGR task would have engaged different ranges of shoulder and elbow angles at each of the targets.

It should be noted that the findings of the present study parallel those of Otaka and colleagues [28], who quantified relationships between outcome measures on the visually guided reach task on the Kinarm with clinical outcomes, including the MAS. Both papers report low-to-moderate correlations between Kinarm outcomes and the MAS; however, Otaka’s group identified statistically significant correlations of varying strength with VGR outcomes other than those reported here. Differences in the proportion of individuals with MAS = 0 between studies (35/70 in the current study, 10/56 in Otaka et al.) could account for these differences.

### Global versus domain-specific deficits in motor function

It is also important to note that the proportion of participants with a “failing” VGR Task Score was higher in the spasticity group and that Task Score was significantly (although modestly) associated with MAS. The Task Score represents a cumulative metric of motor impairment rather than a specific component of impairment. From this perspective, the present findings indicate that individuals with spasticity demonstrate deficits in movement kinematics. In the context of the individual-parameter findings, it may be that movement time and movement speed are among the more important features of motor output in spasticity or that time and speed are important elements of all the tasks included in the assessment. Alternatively, the present findings can also be interpreted as support for previously-reported findings indicating that the MAS does not correlate well with kinematic measures [18] or that spasticity and paresis have different impacts on motor function [28]. Another possibility is that there are features of control unique to spasticity that are not captured in the individual domains included in the VGR task.

### Deficits in proprioception were not more evident in individuals with spasticity

Interestingly, no statistically significant relationships between MAS and APM outcomes were observed, nor were differences between groups observed for any of the APM outcomes. All of the kinematic data for the APM task were derived by having the affected limb passively moved to the targets, requiring the less-affected limb to position match. This specific component of testing was implemented to overcome the obvious issue of having the robot passively move the less-affected limb and then trying to determine whether affected limb matching was poor due to proprioceptive or motoric deficits. In so doing, it was expected that deficits in proprioception would be observed and associated with clinical measures of spasticity.

We note that these findings should not be interpreted as indicating that proprioceptive deficits do not exist in the Spasticity group. In comparison to the healthy normative data, deficits were observed in both motor and proprioception tasks indicating that individuals with stroke have proprioceptive deficits, irrespective of the presence of spasticity. The present findings simply indicate that the deficits of the individuals with spasticity were not necessarily more impactful than the deficits of those without spasticity. From a more general perspective, the observation that a proportion of participants from both groups failed parameters and tasks in both the VGR and APM tasks (Table 2) implies that rather than being purely motoric in nature, deficits in movement control after stroke are also linked to deficits in proprioception. This position is in line with the findings of Dukelow and colleagues [29], who suggested that both motor and proprioceptive deficits are present after stroke, even though they are statistically independent from each other.

Again, the idea of a spasticity zone [27] may explain why proprioception deficits were not observed. In this case, the locations to which the affected limb was passively moved may not have required elbow angular ranges within which spasticity occurred. However, given the observation that participants in the spasticity group were assessed as MAS = 2 or 3, resistance to passive movement would have been detected through most of the range of motion and within workspace covered by the APM task. It is important to consider that the APM task only characterized one component of proprioception – position sense. Other features like kinesthesia (sense of limb motion) or sense of effort also reflect proprioception, but these were not included in the current study. It is possible that although spasticity and position sense are independent from each other, other components of proprioprioception may be more related to spasticity [30].

### Limitations

One measure that is not included here, but which may be a confounder to motor output in spasticity [31, 32] is muscle strength. Because the planar movements that comprise the present study are performed with the limbs supported and because the overall range of movement is relatively small, the potential contribution of impaired strength may be somewhat mitigated. However, strength should be taken into consideration in further understanding the factors that impact motor control in individuals with spasticity. In addition, the only sensory modality that was examined in the present study was proprioception. Recent work has identified kinesthesia as also being impaired after stroke [33, 34]. Kinesthetic deficits may also be a greater determinant of motor function in individuals with post-stroke spasticity or may be more indicative of the types of sensory deficits that occur with spasticity.

Other methodological limitations include the absence of direct measures of proprioception, assessment of spasticity using only one clinical scale, and that we did not record electromyographic activity of muscle during movement. Such direct measures would have provided a more complete characterization of the study cohort and a clearer picture of the existing proprioceptive and muscle state. However the focus of this work was on the kinematic comparison.

One methodological limitation related to recruitment is that only part of the Spasticity cohort were assessed for elbow extensor spasticity. Five individuals with spasticity of both the flexors and extensors were included to balance the group sample sizes to as great an extent as possible. Extensor spasticity was also not assessed on all participants in the No Spasticity group. Thus, it is possible that individuals in the No Spasticity group may have had extensor spasticity, which would have impacted the ability to observe larger differences between groups. The findings could have been more robust with a more homogeneous spastic cohort. This also applies to the possible limitation of the timing of administration of spasticity management interventions at the time of assessment and the extent to which these interventions impacted the ability to identify differences between groups.

## Conclusions

Individuals with and without upper limb spasticity demonstrate deficits in both movement kinematics and proprioception, even months-to-years after their stroke; however, only kinematic deficits are greater in individuals with spasticity. More specifically, measures characterizing temporal features of movement and global measures of movement deficits are most impacted and are also correlated with clinical scores of spasticity (MAS). This work contributes to the growing body of literature characterizing the impact of upper limb spasticity on motor control.

## Data Availability

The data that support the findings of this study are available from the corresponding author upon request.

## Abbreviations

APM: Arm Position Matching
Con/Exp XY: Contraction-Expansion ratio
IDA: Initial direction angle
MAS: Modified Ashworth Scale
MS: Movement speed
MT: Movement time
PLR: Path-length ratio
PS: Posture speed
SMC: Speed maxima count
Var: Variability
VGR: Visually Guided Reaching.
